# Probing the molecular basis of hERG drug block with unnatural amino acids

**DOI:** 10.1038/s41598-017-18448-x

**Published:** 2018-01-10

**Authors:** Logan C. Macdonald, Robin Y. Kim, Harley T. Kurata, David Fedida

**Affiliations:** 1 Department of Anesthesiology, Pharmacology and Therapeutics, University of British Columbia, Vancouver, British Columbia V6T 1Z3 Canada; 2 Department of Pharmacology, University of Alberta, Edmonton, Alberta T6G 2H7 Canada

## Abstract

Repolarization of the cardiac action potential is primarily mediated by two voltage-dependent potassium currents: *I*_*Kr*_ and *I*_*Ks*_. The voltage-gated potassium channel that gives rise to *I*_Kr_, K_v_11.1 (hERG), is uniquely susceptible to high-affinity block by a wide range of drug classes. Pore residues Tyr652 and Phe656 are critical to potent drug interaction with hERG. It is considered that the molecular basis of this broad-spectrum drug block phenomenon occurs through interactions specific to the aromatic nature of the side chains at Tyr652 and Phe656. In this study, we used nonsense suppression to incorporate singly and doubly fluorinated phenylalanine residues at Tyr652 and Phe656 to assess cation-π interactions in hERG terfenadine, quinidine, and dofetilide block. Incorporation of these unnatural amino acids was achieved with minimal alteration to channel activation or inactivation gating. Our assessment of terfenadine, quinidine, and dofetilide block did not reveal evidence of a cation-π interaction at either aromatic residue, but, interestingly, shows that certain fluoro-Phe substitutions at position 652 result in weaker  drug potency.

## Introduction

The *human ether-à-go-go related gene* (hERG) voltage-gated potassium channel is well-known for its repolarizing role in the cardiac action potential as the rapid delayed rectifier potassium current, *I*_Kr_^1,2^. Instances in which hERG function is impaired, through either genetic mutation or drug block, reduce *I*_Kr_ and therefore increase the time needed to repolarize cardiomyocytes^3,4^. On an electrocardiogram, this manifests as a prolongation of the QT interval and is referred to as long QT syndrome type 2 (LQT2)^1,4,5^. LQT2 can have dire consequences such as ventricular tachycardia and sudden death^5^.

LQT2 induced by hERG drug block (acquired LQT2) has received considerable attention over the past twenty years as hERG has been found to be uniquely susceptible to block by a wide array of drugs across multiple pharmaceutical classes^5,6^. Many of these drugs have similar chemical features, such as multiple phenyl rings and a basic amine group^7^, but, many drugs that do not conform to these features also block the channel at therapeutically relevant concentrations, thereby hindering precise predictive tools^8^. As hERG channels bind a wide array of pharmaceuticals in an off-target manner, it is no surprise that many drug-induced arrhythmias stem from acquired LQT2^7^. The significance of this problem has yielded substantial interest in understanding structural features within the channel that give rise to hERG channel blockade. Many studies have highlighted two aromatic residues at the intracellular side of the S6 domain specific to the *eag* family, Tyr652 and Phe656^9–19^. In hERG, substitution of either of these residues to an alanine drastically reduces the potency of most hERG blockers^10^. Experimental and molecular dynamics studies suggest that the aromaticity of these residues underlie the basis of hERG drug block, through either cation-π^5,12,20–22^ or π-stacking interactions^18,23–25^.

Means of investigating potential cation-π relationships in ion channels have been well established^26–29^. Incorporation of fluorinated aromatic amino acid analogues through unnatural amino acid (UAA) mutagenesis allows manipulation of the π-electron density of a specific aromatic residue^30,31^. The electron withdrawing effects of fluorine reduces π-electron density in the centre of the phenyl ring, weakening propensity for cation-π interaction while imposing only subtle steric changes. In this study, we have used fluorinated phenylalanine derivatives to assess whether cation-π interactions are involved in high affinity hERG block by terfenadine, quinidine, and dofetilide. Our data show that unnatural amino acids can be successfully incorporated into hERG with minimal disruption of channel gating, and demonstrate that cation-π interactions do not significantly influence hERG block at the aromatic residues Tyr652 and Phe656.

## Results

### Aromatic amino acids Tyr652 and Phe656 highlighted in past drug block studies

**Figure 1:**
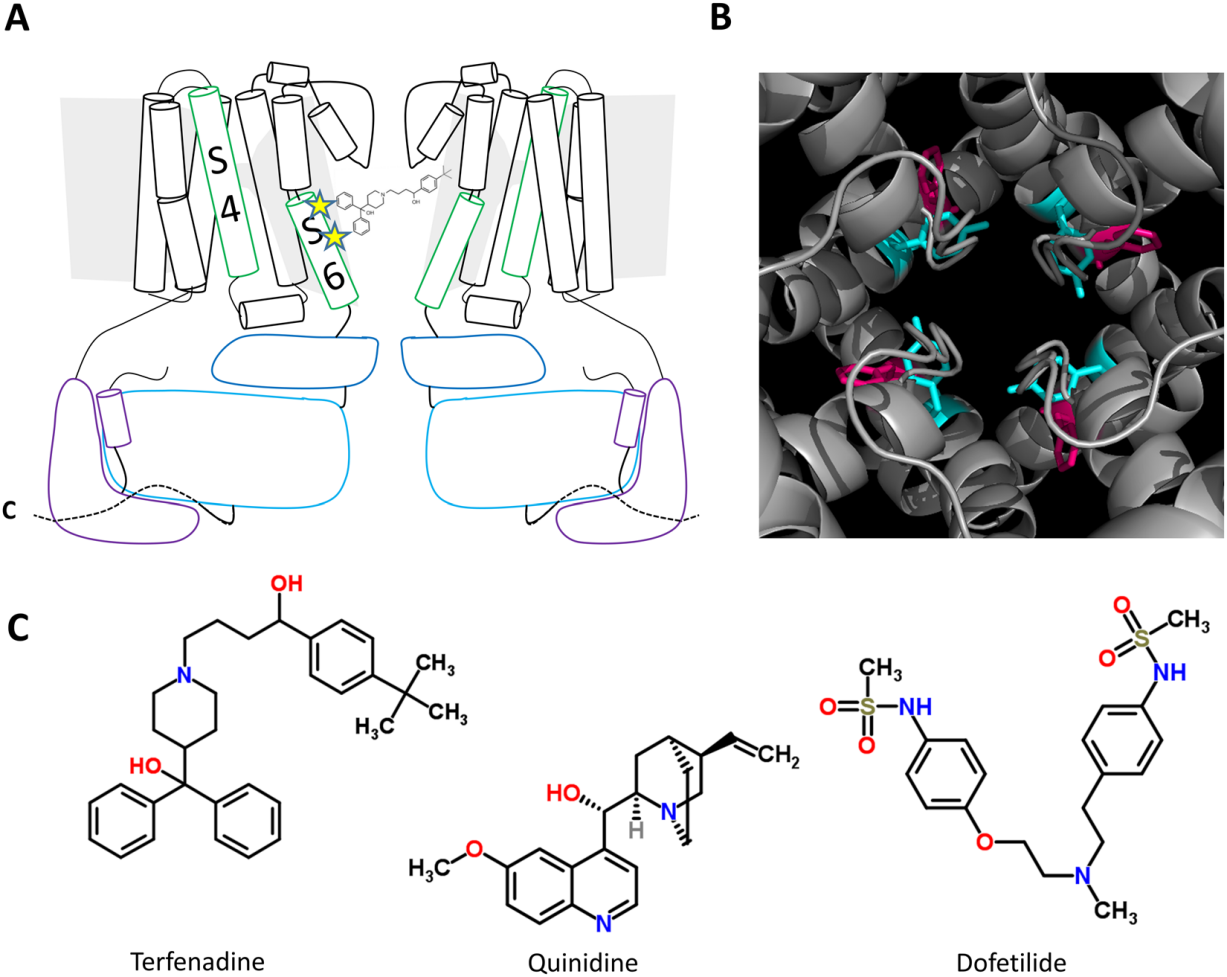
Aromatic residues Tyr652 and Phe656 in hERG shown to be important in drug block. (**A**) Cartoon schematic of approximate location of Tyr652 and Phe656 in hERG pore region. (**B**) Top-down view of the pore region of hERG pore from cryo-EM structure 5VA1^32^. Aromatic residues Tyr652 and Phe656 are highlighted in blue and pink respectively. (**C**) Chemical structures of terfenadine, quinidine, and dofetilide.

Figure 1A is a schematic of two of the four subunits of the hERG channel structure. The approximate locations of Tyr652 and Phe656, previously shown to underlie high affinity drug block of the hERG channel, are highlighted as stars^9–19^. Figure 1B gives a more detailed view of the pore region of hERG structure from the cryo-EM structure of the open state of the channel^32^. Residue Tyr652 is highlighted in blue and Phe656 is highlighted in pink. As seen in the structure, residues Tyr652 and Phe656 are roughly one helical turn from one another and their aromatic side chains are easily accessible from the pore region. The aromaticity of these residues has led to the suggestion that cation-π interactions may be an important factor leading to the non-selective drug block phenomenon seen in hERG. Figure 1C shows the structures of the drugs assessed in this study: terfenadine, quinidine, and dofetilide.

### Incorporation of UAA into K_v_ 11.1

Testing whether a cation-π relationship occurs at an aromatic residue can be accomplished by measuring the impact of substitution with a series of fluorinated aromatic amino acids. Successive fluorinations of aromatic side chains yield additive reductions in ability to donate π electrons to cation-π interactions^30^. Through the use of nonsense suppression, we incorporated singly and doubly fluorinated phenylalanine derivatives at both Tyr652 and Phe656. These UAA also have the advantage of providing a sterically conservative means of studying the specific nature of interactions of individual residues. If hERG drug interactions depend largely on cation-π binding interactions at sites Tyr652 and Phe656, then successive fluorinations at these sites should decrease drug affinity in a linear manner^30^. Several past studies have successfully described cation-π relationships using this method^27,28,33,34^.

### Fluorinated phenylalanine derivatives are well tolerated at Tyr652 and Phe656

**Figure 2:**
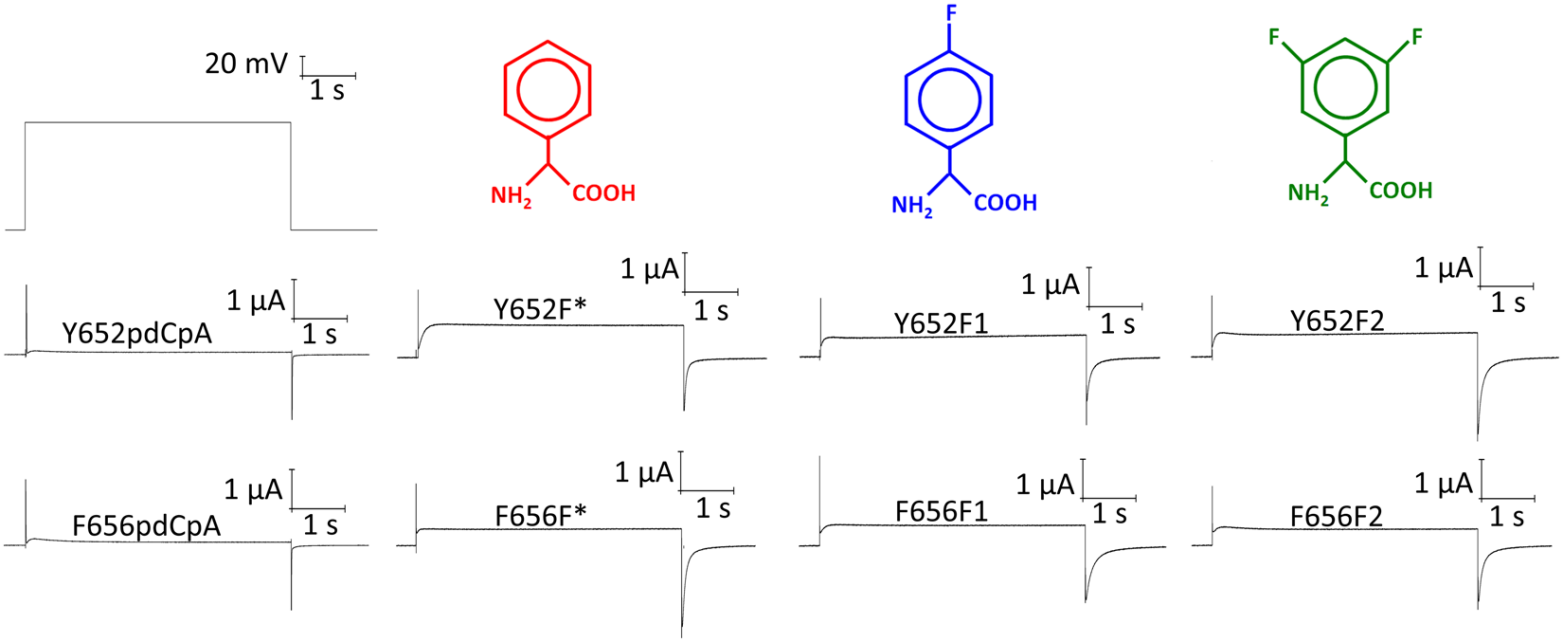
Incorporation of natural and unnatural amino acids through nonsense suppression is well tolerated at both Tyr652 and Phe656. A 5 s depolarization to 0 mV from a holding potential of −110 mV was used to assess construct expression. All mutants were successfully expressed via nonsense suppression and representative traces are displayed here.

Figure 2 shows representative hERG current traces from all mutants in two electrode voltage clamp experiments. In experiments in which tRNA was not appended to an amino acid, current levels do not rise above a 0.2 μA tail following a 5 s depolarization to 0 mV, which we attribute to an endogenous current. The UAA are well tolerated in terms of maintaining a hERG-like phenotype. The unnaturally incorporated phenylalanine is termed Y652F* or F656F*, the singly fluorinated phenylalanine incorporation is termed Y652F1 or F656F1, and the doubly fluorinated phenylalanine incorporation is termed Y652F2 or F656F2. Currents elicited from this protocol are small during depolarisation as channels that slowly open quickly enter an inactivated state, but upon repolarisation to −110 mV, a hooked tail current is observed for all of the mutant channels. This reflects relief from inactivation followed by deactivation. Expression varied considerably amongst the eggs injected with UAA. As such, we required that expression reached an arbitrary level of 0.5 µA at −50 mV for the data to be included in the analysis. In our assessment of drug block, a step to −50 mV after a prolonged activation was used to measure the level of drug block. We assessed the parameters of steady state voltage-dependence of activation and steady-state voltage-dependence of inactivation for each mutant construct.

### Voltage-dependence of activation

**Figure 3:**
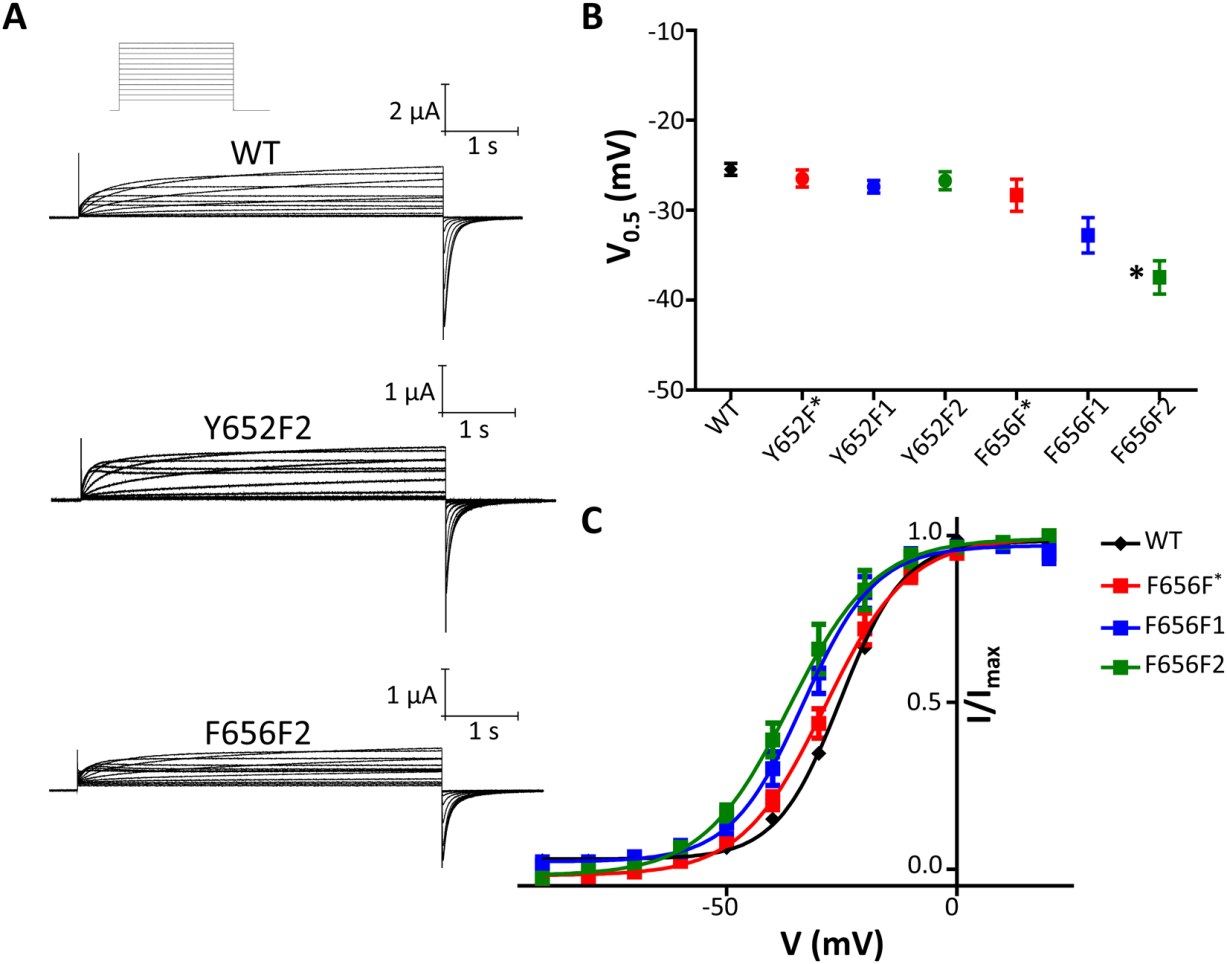
Voltage-dependence of activation is unchanged by Phe fluorination at position 652, but hyperpolarized at position 656. (**A**) Ionic current from WT, Y652F2, and F656F2 mutant hERG channels recorded during 5 s depolarizations from −110 mV to 20 mV in 10 mV increments followed by repolarization to −110 mV (protocol inset at top). Pulses were applied every 7 s. (**B**) GV_0.5_ values for all constructs. GV fit parameters are found in Table S1. (**C**) GV relationships of the UAA mutants at position 656 and WT as measured from tail currents at −110 mV.

Figure 3A shows representative traces from WT, Y652F2, and F656F2 elicited from a protocol (inset) used to assess the voltage-dependence of activation (GV). The phenylalanine mutation at position Tyr652 had minimal effect on the steady-state GV. Similarly, as can be seen in Fig. 3B, the fluorinated Tyr652 mutants resulted in only a minimal change in steady-state GV. Figure 3B shows that upon increasing fluorination of Phe656, a hyperpolarizing left shift in the GV occurs. Significant differences in the figure are denoted by stars (*P < 0.05). Figure 3C shows the Boltzmann curve fits for the Phe656 series of fluorinated phenylalanine mutants and their hyperpolarizing shifts upon increased fluorination at Phe656. Table S1 displays the V_0.5_ and equivalent charge for all constructs.

### Voltage-dependence of inactivation unchanged for all constructs

**Figure 4:**
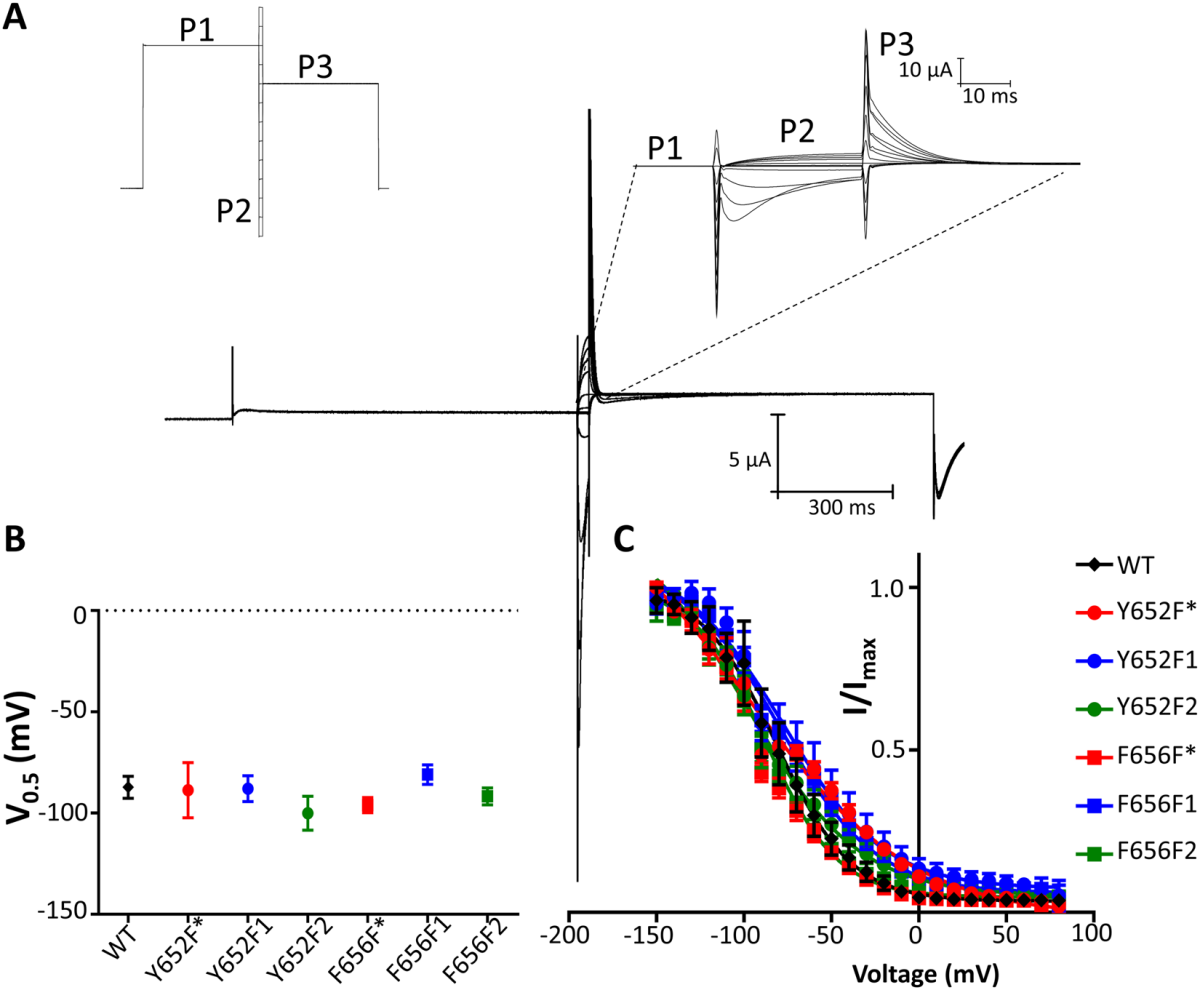
Voltage-dependence of inactivation unchanged among mutant channels. (**A**) Representative trace of WT hERG current resulting from the inactivation protocol (inset left). The protocol is described in further detail in Methods. An expanded view of the currents during the P2 and P3 pulses are shown in the right inset. (**B**) V_0.5_ values for inactivation of all constructs. Fit parameters are found in Table S1. (**C**) Steady-state inactivation relationships of all constructs.

Several past studies have suggested that drugs that block hERG have a selective preference for binding to the inactivated state of the channel^35–38^ and thus, it was important to measure inactivation properties of the mutants. The steady-state voltage-dependence of inactivation was assessed by a triple pulse protocol as shown in the inset of Fig. 4A. The first pulse is a 900 ms depolarization to +40 mV that opens and inactivates hERG channels. This is followed by a short 30 ms pulse to potentials from −150 to +80 in 10 mV increments, allowing channels to reach a steady-state of inactivation with minimal contamination of the signal from channels entering a deactivated state. The third pulse is to 0 mV and the resulting instantaneous tail currents at the beginning of this pulse from each sweep can be normalized to the maximum value and then fit with a Boltzmann function, as described. The inset in Fig. 4A shows that the capacitive transient at the beginning of the third pulse lasts no longer than 1 ms, allowing assessment of the proportion of inactivated channels. The measured V_0.5_ values in Fig. 4B show that the steady-state voltage-dependence of inactivation did not vary considerably amongst mutant channels and was not statistically different for any of the mutant channels when compared to the WT channel using a Dunnett post-test. Figure 4C shows Boltzmann fits to the steady-state inactivation-voltage relationships. Table S1 shows the V_0.5_ and equivalent charge for all constructs.

### No evidence of a cation-π relationship detected for hERG terfenadine block at Tyr652 or Phe656

**Figure 5:**
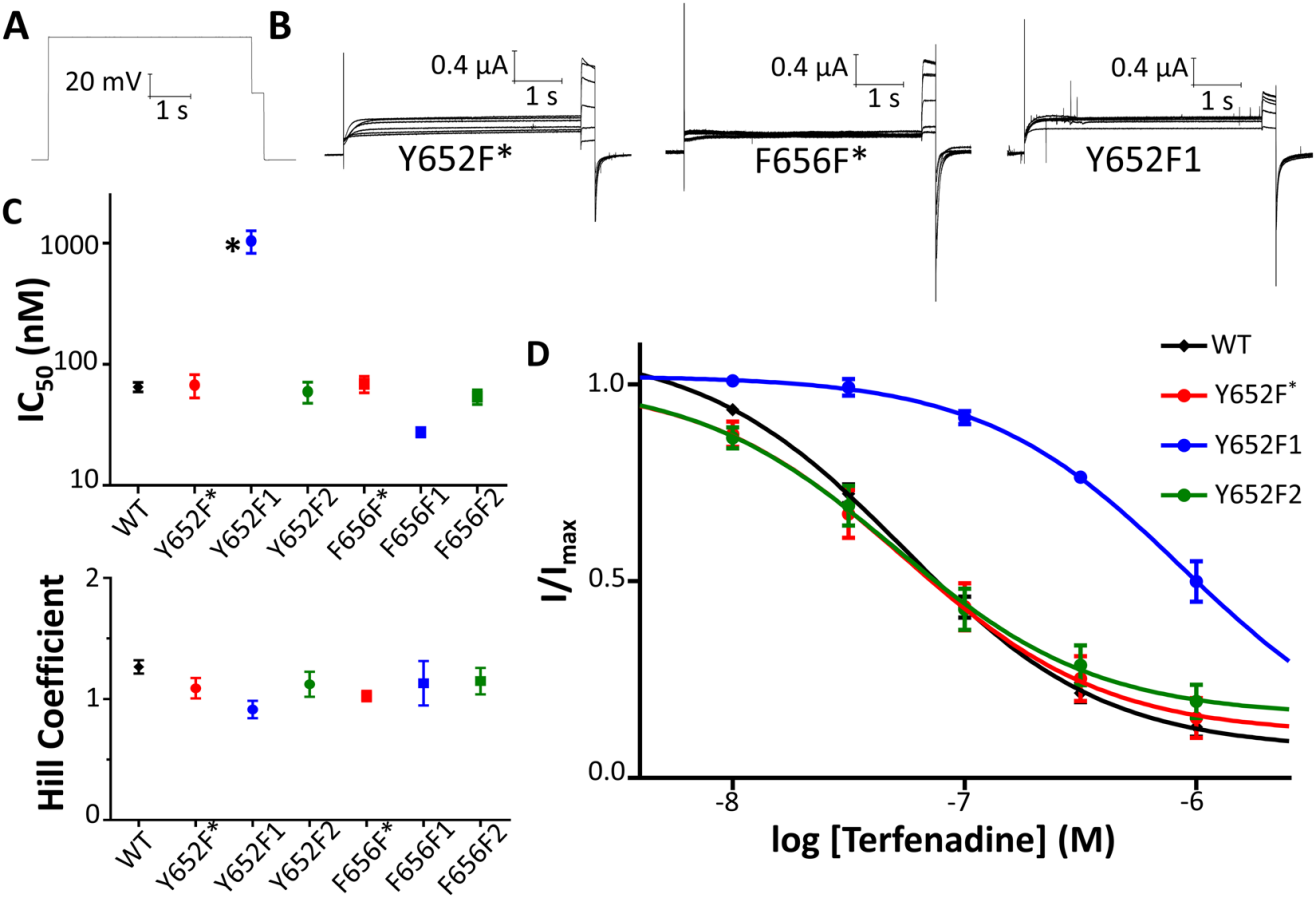
Fluorination of Phe656 does not reduce terfenadine potency; however, mono-fluorination of Y652F at the C4 phenyl ring carbon results in a large reduction in terfenadine potency. (**A**) Protocol used to evaluate drug block. From a holding potential of −110 mV, channels are depolarized to 0 mV for 5 s and are then repolarized first to −50 mV for 300 ms and then back to −110 mV. (**B**) Currents from Y652F*, F656F*, and Y652F1 in control and in response to terfenadine concentrations of 10, 30, 100, 300, 1000 nM. (**C**) Terfenadine IC_50_s and Hill coefficients. IC_50_ and h fit parameters are found in Table S2. (**D**) Concentration-response curves of WT and Y652F series.

Figure 5A shows the protocol used to assess drug block. This protocol is repeated in successive sweeps as either control or drug-containing solutions flow into the bath chamber and over the oocyte. A higher concentration of drug is not started until the last concentration of drug has reached a steady state level of block. Figure 5B shows representative traces of constructs Y652F*, F656F*, and Y652F1 in solutions of no drug and in solutions with terfenadine concentrations of 10 nM, 30 nM, 100 nM, 300 nM, and 1 μM. Upon introduction of 10 nM terfenadine, minimal block is observed at −50 mV, but upon introduction of 30 nM terfenadine the amplitude of the current at −50 mV becomes observably decreased. Higher concentrations of terfenadine lead to further reductions in current. The steady state current at −50 mV reached at each drug concentration was normalized to the steady state current at −50 mV with no drug. From these normalized values, a concentration-response curve was generated and fit with a Hill equation. IC_50_ and Hill coefficient values were generated from each Hill fit. Figure 5C shows the IC_50_ and Hill coefficients of all mutant channels with respect to terfenadine block. Values are shown in Table S2. The IC_50_ for the Phe656 mutants did not change significantly upon increased fluorination of the phenylalanine. The IC_50_ is greatly increased for the singly fluorinated Tyr652 mutant, but not the doubly fluorinated mutant. It is only when the C4 carbon of the phenylalanine is fluorinated that reduced potency is seen. This is not what would be expected from a cation-π relationship. Figure 5D shows the concentration response curves for the Tyr652 fluorinated phenylalanine mutants. The curve for the Y652F1 mutant is greatly right-shifted compared to the WT hERG, Y652F*, and Y652F2 constructs.

### No evidence of a cation-π relationship detected for hERG quinidine block at Tyr652 or Phe656

**Figure 6:**
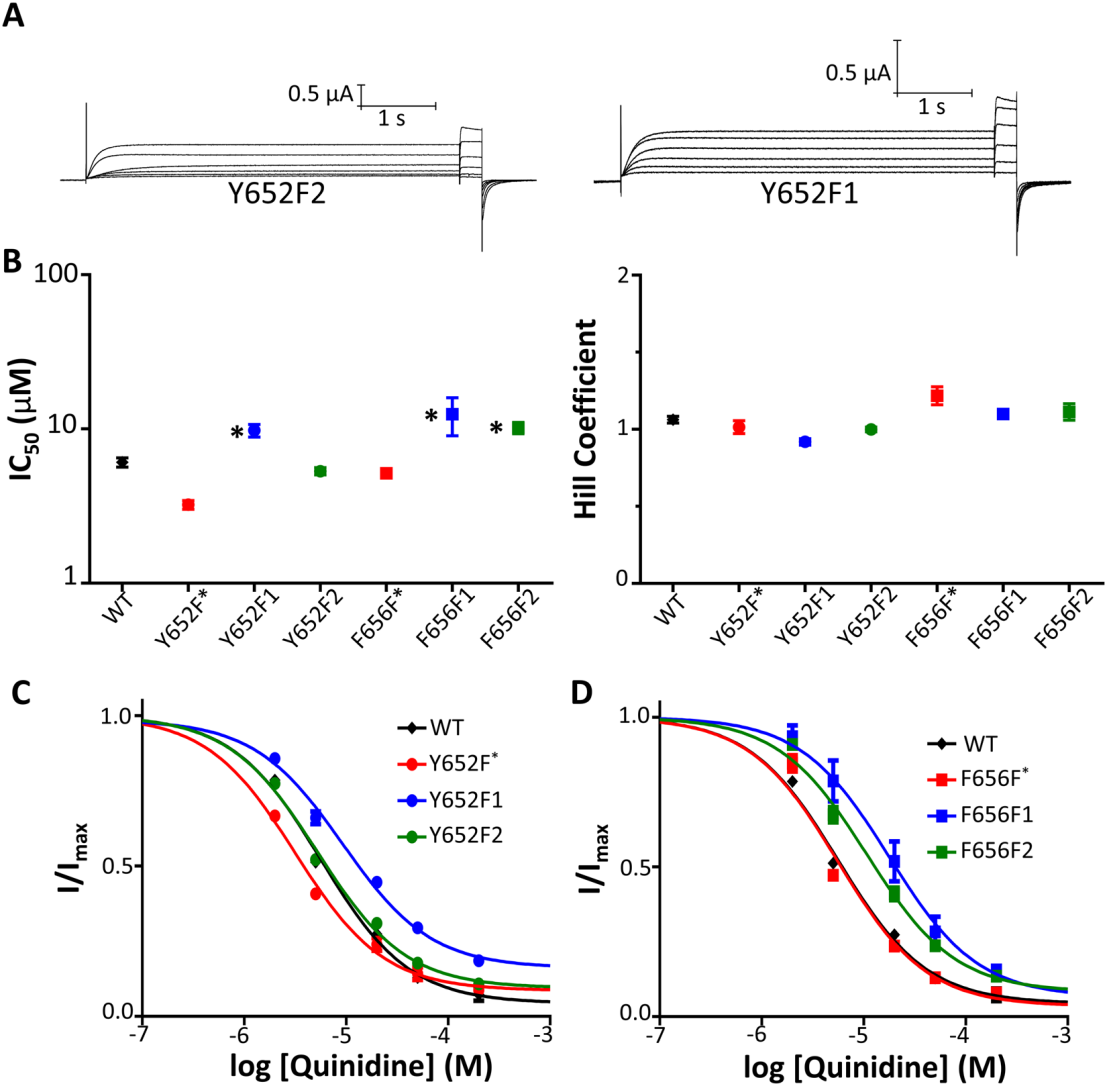
Fluorination of Phe656 reduces quinidine potency in a non-additive fashion; however, only mono-fluorination of Y652F at the C4 phenyl ring carbon results in reduced quinidine potency. (**A**) Currents from Y652F2 and Y652F1 elicited from the protocol shown in Fig. 4A in control and in response to quinidine concentrations of 2, 6, 20, 60, 200 μM (**B**) Quinidine IC_50_ and Hill coefficients. IC_50_ and h fit parameters are found in Table S2. (**C**) Concentration-response curves of Y652F fluorinated mutant series and WT (**D**) Concentration-response curves of Phe656 fluorinated mutant series and WT.

Figure 6A shows representative current traces from constructs Y652F2 and Y652F1 in solutions of no drug and in solutions with quinidine concentrations of 2 μM, 6 μM, 20 μM, 60 μM, and 200 μM. The steady-state current at −50 mV is progressively reduced in higher concentrations of quinidine. Figure 6B shows the IC_50_ relationships and Hill coefficients of all mutant channels with respect to quinidine block. Values are shown in Table S2. At Tyr652, as seen with the terfenadine data, fluorination only reduced potency when the fluorine was incorporated in the C4 carbon position. The F2 mutant was again unaffected by fluorination as compared to WT. Again, this is not consistent with what is expected if cation-π interactions were a primary determinant of drug binding. The Phe656 fluorinated phenylalanine constructs both exhibited a significant decrease in potency to the same degree upon any level of fluorination. Figure 6C and D show the concentration response curves for Tyr652 and Phe656 families of fluorinated phenylalanine mutants, respectively.

### No evidence of a cation-π relationship detected for hERG dofetilide block at Tyr652 or Phe656

**Figure 7:**
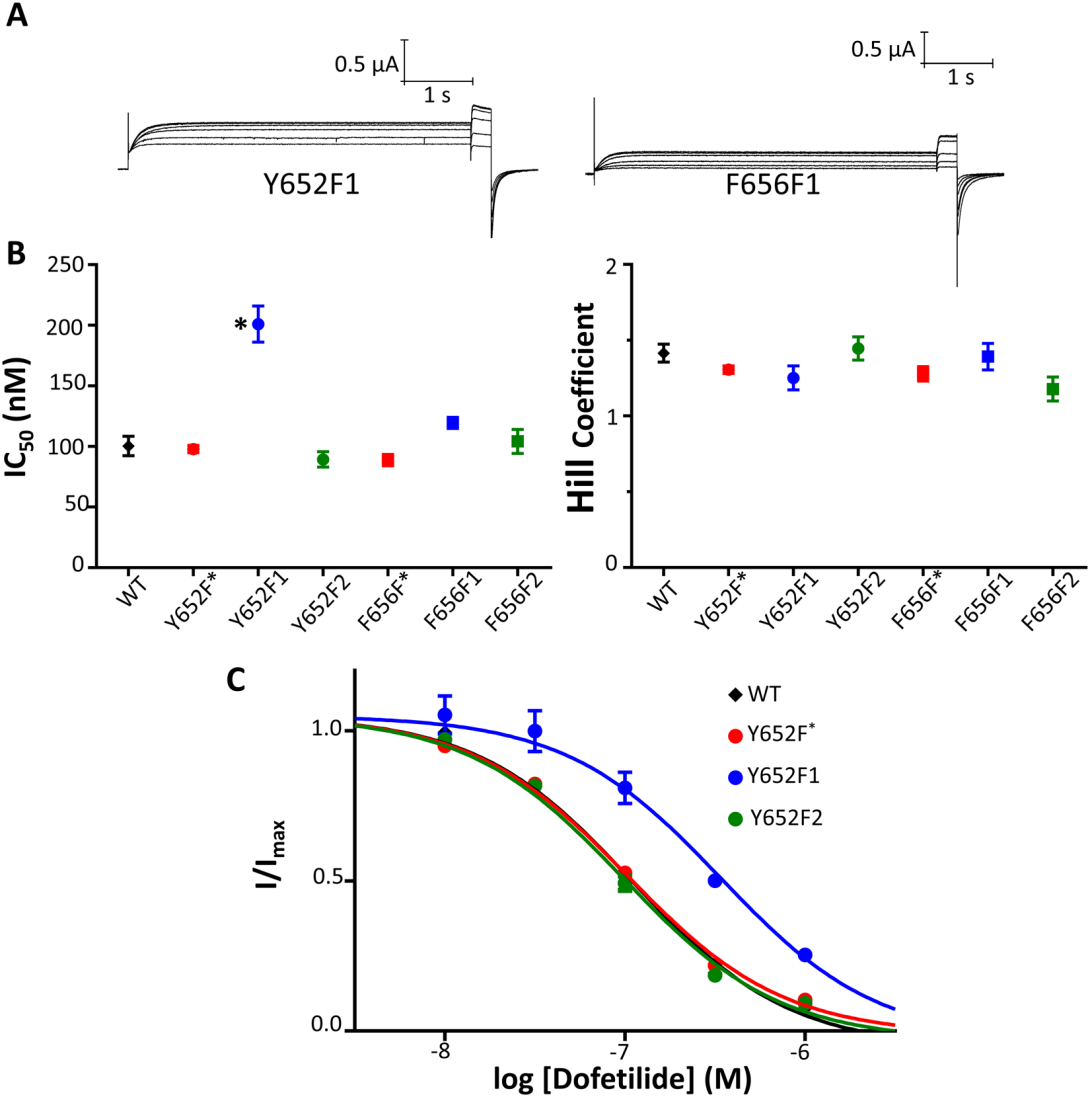
Fluorination of Phe656 does not reduce dofetilide potency; however, mono-fluorination of Y652F at the C4 carbon of the phenyl ring results in a large reduction in dofetilide potency. (**A**) Currents from Y652F1 and F656F1 in control and in response to dofetilide concentrations of 10, 30, 100, 300, 1000 nM. (**B**) Dofetilide IC_50_ and Hill coefficients. IC_50_ and h fit parameters are found in Table S2. (**C**) Concentration-response curves of WT and Y652F series.

Figure 7A shows representative traces of constructs Y652F1 and F656F1 in solutions of no drug and dofetilide concentrations of 10 nM, 30 nM, 100 nM, 300 nM, and 1 μM. Current at −50 mV is progressively reduced at higher concentrations of dofetilide. Figure 7B shows the IC_50_ relationships and Hill coefficients of all mutant channels with respect to dofetilide block. IC_50_ and Hill coefficient values are shown in Table S2. Similar to what was seen with terfenadine, fluorination had minimal effect on potency of dofetilide block at position Phe656. Also similar to what was seen with both terfenadine and quinidine, fluorination of the C4 carbon for the Tyr652 fluorinated phenylalanines reduced potency, but Y652F2 potency was unaffected (similar to WT and Y652F*).

## Discussion

The hERG channel presents a major hurdle in the drug development process due to its penchant for drug block. The main determinants of drug interaction in hERG are two aromatic residues in the S6 transmembrane segment: Tyr652 and Phe656^7,10,16^. The aromaticity of these two residues has led to suggestion that the basis of this generalized drug interaction is due to molecular interactions specific to the π-electrons of aromatic systems^5,12,18,20–25^. Through two electrode voltage clamp experiments and UAA mutagenesis, we attempted to determine the extent to which cation-π drug-protein interactions play a role in the molecular basis of hERG drug block at Tyr652 and Phe656. Our experiments have found no indication that cation-π interactions play a significant role in hERG drug block at either Tyr652 or Phe656.

Several past studies have suggested that a variation of the modulated receptor hypothesis^39^ may apply to the hERG channel^7,35–38^. These studies have noted that some drugs that block the hERG channel seem to preferentially interact with the inactivated state(s) of the channel^35–38^, although this effect is not fully understood^38^. The three drugs investigated in our study have been shown by Yang *et al*. in^36^ to exhibit inactivation-dependent block^36^. This study found that by introducing the S631A mutation which abolishes inactivation, the potency of dofetilide, terfenadine, and quinidine were all reduced. As such, we sought to assess whether the constructs used in our experiments affected the voltage-dependence of inactivation as any large change in this parameter could have affected the interpretation of our drug block data.

Our data (Fig. 4) show no significant differences in the voltage-dependence of inactivation are produced by the unnatural substitutions, ruling against the possibility that changes in drug potency are caused by alterations in channel inactivation properties. To be especially noted is that the steady-state inactivation V_0.5_ of Y652F1, the only mutant in our study to have large reductions in potency, is unchanged from that of WT and Y652F*.

We also determined the GV relationships for all of our constructs (Fig. 3). The fluorinated Tyr652 series of constructs showed no change in the GV. At Phe656 there was a hyperpolarizing shift of the GV curve upon increasing fluorination of the aromatic ring. The V_0.5_ of activation shifted from −28.3 ± 1.8 mV for F656F*, to −32.8 ± 2.0 mV for F656F1, and to −37.5 ± 1.9 mV for F656 F2. The change in this parameter is unlikely to have an effect on our ability to accurately measure drug block of the channel as our voltage protocol to assess drug block involves an initial 5 s depolarization to 0 mV, which would be sufficient to bring all constructs to equilibrium close to maximum conductance. We did not pursue this phenomenon further in this study as the purpose of this work was to investigate the molecular basis of drug block at Tyr652 and Phe656.

Our data show that cation-π interactions do not play a role at either Tyr652 or Phe656 for hERG block by terfenadine, quinidine, or dofetilide (Figs 5, 6 and 7) in the steady state. It should be noted that our data do not address cation-π involvement in intermediate steps of drug binding, as we did not obtain onset kinetics for the block process in oocytes. Should a cation-π interaction take place at either site, then increasing fluorination of the phenyl ring should cause a progressive increase in IC_50_, indicating a reduced affinity of the drug for the binding site^30^. Interestingly, the potencies of all drugs studied, as reflected by IC_50_ values, were initially decreased with single fluorination of the phenylalanine derivatives at Tyr652. However, upon doubly fluorinating the phenylalanine derivatives, the potency of the drug interaction remained unchanged compared to the WT channel. This is a stark contrast to the effect of doubly fluorinating Trp-149 in the α-subunit of the nicotinic receptor (Zhong *et al*.^31^), which reduces acetyl choline potency by nearly an order of magnitude. As shown in Fig. 2, the site of fluorine incorporation in the F1 and F2 phenylalanine derivatives are on different carbons of the phenyl ring. The F1 fluorine is incorporated at the C4 position, whereas the F2 fluorines are incorporated at the C3 and C5 positions. The C4 position appears to be important to an interaction that is weakened upon introduction of a fluorine to this position. The possibility exists that the fluorine incorporation at the C4 position introduces changes to the electrostatic potential of the phenyl ring in a way that disrupts the π-stacking relationship. However, in the WT channel the native tyrosine residue would have an alcohol group at the C4 position. This makes it unlikely that non-polarity of that specific region of the phenyl ring is important for high-affinity drug block. The mutant channel Y652W also conserves the potency of drug block and can even reintroduce drug block in non-inactivating constructs^12,40^. Many *in silico* molecular dynamics studies show that Tyr652 is involved in π-stacking interactions during hERG drug block^18,23–25^. Substituent effects on π-stacking interactions are still under debate and, as such, it is difficult to conclude much about the impact of our unnatural substitutions on this potential relationship of drugs with Tyr652. Alternatively, should drug interactions at Tyr652 largely be coordinated through hydrophobic interactions, then polarization of the ring could potentially decrease drug affinity. It would have to be a very site-specific hydrophobic interaction, though, as the doubly fluorinated phenylalanine derivative did not change drug affinity.

Another possibility is the singly fluorinated UAA introduces a steric change in the orientation of the pore region that makes it less favourable for drug interaction. We consider this to be unlikely because of the subtlety of the substitution. However, the increased polarization of the ring upon C4 fluorination would increase the hydrophilicity of the structure, but whether or not this would be enough to effect larger changes in pore structure would only be speculation. All that can be said conclusively about our introductions of fluorinated phenylalanine derivatives at Tyr652 is that our results are not supportive of a cation-π interaction.

Drug potency in experiments involving fluorinated phenylalanine derivatives incorporated at Phe656 was not affected in terfenadine or dofetilide drug block. In experiments involving quinidine, potency was reduced upon any degree of fluorination. Neither of these are indicative of a cation-π interaction.

Recently, the open state structure of hERG was determined by cryo-EM^32^. The authors of this study noted the presence of unique hydrophobic pockets in the central cavity, components of which include residues Tyr652 and Phe656. It may be that hydrophobic interactions and not interactions specific to the aromatic nature of Tyr652 or Phe656 are the primary means of drug interaction with hERG. This would fit well with findings that drug block potency correlates well with hydrophobic amino acids at these sites^12^, though this study did suggest that an aromatic amino acid was necessary for high potency block at residue 652.

In summary, our data do not uncover evidence of cation-π activity at either Tyr652 or Phe656 in the steady state. This study contributes significantly to the understanding of hERG drug block as it provides experimental data in support of previous molecular dynamic drug docking studies that have argued against cation-π activity being a large contributor to hERG drug block^18,23,24^.

## Materials and Methods

### Molecular Biology

Incorporation of UAA through nonsense suppression was performed as described^26^. UAAs were coupled to nitroveratryloxycarbonyl as a protecting group and activated as the cyanomethyl ester. This was then coupled to pdCpA (GE Healthcare/Dharmacon, Lafayette, CO), an aminoacyl dinucleotide, which was then ligated to a modified (G73) *Tetrahymena thermophile* tRNA. The amino-acylated tRNA-UAA was de-protected via ultraviolet irradiation immediately before oocyte injection.

Site-directed mutagenesis of WT hERG DNA in a pBluescript SK+ vector was used to make the Y652TAG and F656TAG constructs with the QuikChange II system (Stratagene). Mutations were confirmed by direct sequencing (Macrogen). DNA was linearized with *NotI* prior to RNA transcription using the mMessage mMachine T7 Ultra transcription kit (Ambion).

### Oocyte Preparation and Injection

*Xenopus laevis* oocytes were used for two electrode voltage clamp experiments. Mature female *Xenopus laevis* frogs (Boreal Science) were anaesthetized in a 2 g/L tricaine methanesulfonate solution at pH 7.4. Under anaesthesia, the animal was euthanized in accordance with University of British Columbia animal care protocols. The ovarian lobes were extracted and divided into sections of 10–20 eggs before undergoing follicular layer digestion for 1–2 hours in a 1 mg/mL collagenase A calcium-free solution (82.5 mM NaCl, 2.5 mM KCl, 1 mM MgCl_2_, 5 mM HEPES buffer, pH adjusted to 7.6). The digested eggs were washed in calcium-free solution and stored in OR3 media (500 mL Liebovitz’s L-15 medium, 15 mM HEPES, 1 mM glutamine, 500 μM gentamycin, made up to 1 L with distilled water and pH adjusted to 7.6). Stage IV and V oocytes were selected and stored at 18 °C prior to use. Selected oocytes were injected with roughly 80 ng of tRNA-UAA and 40 ng of mutant hERG cRNA in a 50 nL volume. In control experiments, the cRNA alone or the cRNA together with a tRNA coupled to pdCpA (lacking an appended amino acid) were injected.

### Electrophysiology

Two electrode voltage clamp experiments were performed 1–3 days following injection. Experiments were performed using an Oocyte Clamp OC-725C (Warner Instruments) via an Axon Digidata 1440A A/D converter (Molecular Devices) controlled by pClamp10 software (Molecular Devices). In experiments, oocytes were bathed in a continuous flow of ND96 media (96 mM NaCl, 3 mM KCl, 1 mM MgCl_2_, 5 mM HEPES, 2 mM CaCl_2_, pH adjusted to 7.4). All chemicals were obtained from Sigma-Aldrich Chemical Co. (St. Louis, MO). Borosilicate glass was used to pull microelectrodes of 0.1 to 1.0 MΩ resistances using a P-97 Flaming/Brown Micropipette Puller (Sutter Instruments) and filled with 3 M KCl. Drugs were applied with a manual perfusion system. Stock solutions of 10 mM terfenadine, 10 mM dofetilide, and 100 mM quinidine in DMSO were stored at 4 °C and experimental solutions were obtained through dilutions of these stocks on the day of experiments. In control experiments in which a UAA-coupled tRNA was not co-injected, endogenous currents and non-selective incorporation at our site of interest were, at their largest, not found to exceed 0.2 μA tail currents at −50 mV following a prolonged depolarization. Recordings with initial tail currents of less than 0.5 μA at −50 mV following a prolonged activation were discarded. Currents were allowed to reach a stable level before beginning experimental recordings. The holding potential for all experiments was −110 mV. Recordings were performed at room temperature (20–22 °C).

### Data Analysis

Conductance voltage relationships which were obtained by measuring tail currents at −110 mV following 5 s depolarizations to a range of potentials from −110 mV to +40 mV. Data points were normalized to their maximum values and then fit with a Boltzmann function of the form G/G_max_ = 1/(1 + exp(−zF(V − V_0.5_)/RT)).

Steady–state inactivation for all mutants was determined through a triple pulse protocol. The initial 900 ms pulse to +40 mV opens and inactivates nearly all channels present. The second pulse is a short 30 ms pulse to potentials from −150 to +80 in 10 mV increments, allowing channels to inactivate with minimal contamination of the signal from channels entering a deactivated state. The third pulse is to 0 mV and the resulting instantaneous tail currents at the beginning of this pulse from each sweep can be normalized to the maximum value and then fit with a Boltzmann function, as described. For the five most hyperpolarized pulses, it was necessary to account for the deactivation that takes place over the 30 ms pulse. In a protocol run before measuring steady state inactivation, time constants of deactivation were obtained by activating channels with a 900 ms +40 mV pulse, followed by hyperpolarizing pulses to potentials ranging from −150 mV to 0 mV. The kinetic data were fit with single exponential functions of the form G = G_0_ + A *e* ^−t/*τ*^ where G is the normalized response, A is the amplitude, and *τ* is the time constant, or double exponential functions of the form G = G_0_ + A_1_ *e* ^−t/*τ*1^ + A_2_ *e* ^−t/*τ*2^, where A_1_ and A_2_ are the amplitudes of each component of the fit and *τ*1 and *τ*2 the accompanying time constants. These time constants of deactivation were used to adjust for deactivation our steady-state inactivation protocol.

Drug block experiments were performed by a 5 s depolarization to 0 mV followed by a repolarization to −50 mV to remove inactivation and generate a larger signal from which to assess current size. The holding potential used was −110 mV. Drugs were applied in increasing concentrations and allowed to reach a steady state before applying the next higher concentration. Half-maximal inhibition was estimated by fitting the normalized currents (I_drug_/I_control_) with a Hill equation of the form y = A1 + ((A2 − A1)/1 + 10 ^(log K^_A_^− [L])*h)^, where K_A_ is the ligand concentration which results in half maximal block, A refers to asymptotic values, [L] refers to the concentration of drug, and h refers to the Hill coefficient.

Data are presented as mean ± standard error of the mean. F and R have their usual thermodynamic meanings and T = 293.15 K. Graphpad Prism was used to perform one-way analysis of variance with a Dunnett post-test to compare WT with mutant channel values with a significance level set at *P* < 0.05.

### Data Availability

The datasets generated during the current study are available from the corresponding author on request.

## Electronic supplementary material

Supplemental Tables
