# Supplementary material for: Probing the molecular basis of hERG drug block with unnatural amino acids
Source: Sci Rep. 2018 Jan 10;8:289. doi: 10.1038/s41598-017-18448-x (PMC5762913; doi:10.1038/s41598-017-18448-x)
Supplement: Supplementary file 1 — Supplemental Tables [file 41598_2017_18448_MOESM1_ESM.doc]

**Supplementary Information: Probing the molecular basis of hERG drug block with unnatural amino acids**

**Logan C. Macdonald^1^, Robin Y^2^. Kim, Harley T. Kurata^2^, and** [**David Fedida**](http://jgp.rupress.org/search?author1=David+Fedida&sortspec=date&submit=Submit)**^1*^**

^1^ Department of Anesthesiology, Pharmacology and Therapeutics, University of British Columbia, Vancouver, British Columbia V6T 1Z3, Canada.

^2^ Department of Pharmacology, University of Alberta, Edmonton, Alberta T6G 2H7, Canada.

|  | Activation | | | Inactivation | | |
| --- | --- | --- | --- | --- | --- | --- |
| Constructs | V_0.5_ (mV) | z | n | V_0.5_ (mV) | z | n |
| WT | -25.5 ± 0.7 | 3.8 ± 0.1 | 9 | -87.2 ± 5.4 | 1.2 ± 0.2 | 7 |
| Y652F* | -27.0 ± 0.6 | 3.2 ± 0.2 * | 15 | -88.7 ± 13.6 | 0.6 ± 0.1* | 5 |
| Y652F1 | -27.4 ± 0.7 | 2.3 ± 0.1* | 8 | -88.0 ± 6.4 | 0.9 ± 0.1 | 7 |
| Y652F2 | -26.7 ± 1.0 | 3.1 ± 0.1 * | 11 | -100.1 ± 8.4 | 0.8 ± 0.0 | 5 |
| F656F* | -28.3 ± 1.8 | 3.0 ± 0.2 * | 10 | -96.1 ± 3.7 | 1.1 ± 0.1 | 6 |
| F656F1 | -32.8 ± 2.0 | 3.2 ± 0.1 * | 7 | -81.0 ± 4.8 | 0.9 ± 0.1 | 4 |
| F656F2 | -37.5 ± 1.9 * | 3.2 ± 0.2 * | 9 | -91.7 ± 4.2 | 1.2 ± 0.2 | 6 |

**TABLE S1. Activation and inactivation parameters for WT and mutant constructs.** Values are mean ± standard error of the mean. Data are compared using one-way ANOVA with a Dunnett post-test, using WT hERG as control. *P* < 0.05 denoted by asterisks (*).

|  | Terfenadine | | |  | Quinidine |  |  | Dofetilide |  |
| --- | --- | --- | --- | --- | --- | --- | --- | --- | --- |
| Constructs | IC_50_ (nM) | h | n | IC_50_ (μM) | h | n | IC_50_ (nM) | h | n |
| WT | 65± 6 | 1.3± 0.1 | 7 | 6.1± 0.4 | 1.1± 0.0 | 4 | 100 ± 8 | 1.4 ± 0.1 | 5 |
| Y652F* | 67± 15 | 1.1± 0.1 | 7 | 3.2± 0.2 | 1.0± 0.0 | 6 | 98± 3 | 1.3± 0.1 | 4 |
| Y652F1 | 1040 ± 350 * | 1.0 ± 0.3 | 3 | 9.8± 0.9 * | 0.9± 0.0 | 6 | 200± 15* | 1.3± 0.1 | 6 |
| Y652F2 | 59 ± 12 | 1.1± 0.1 | 8 | 5.3± 0.3 | 1.0± 0.0 | 8 | 90 ± 6 | 1.4± 0.1 | 4 |
| F656F* | 69 ± 11 | 1.0± 0.0 | 4 | 5.2± 0.2 | 1.3± 0.2 | 5 | 89 ± 5 | 1.3± 0.0 | 6 |
| F656F1 | 28± 3 | 1.1± 0.2 | 4 | 16 ± 4.0* | 1.1 ± 0.1 | 3 | 119± 5 | 1.4± 0.1 | 4 |
| F656F2 | 54± 7 | 1.1± 0.1 | 4 | 10.2± 0.9* | 1.1± 0.1 | 6 | 104± 10 | 1.2± 0.1 | 3 |
| Cation – π behaviour? | No | | | No | | | No | | |

**TABLE S2. Concentration of half maximal block and Hill coefficient for WT and all mutant constructs.** Values are mean ± standard error of the mean. Data are compared using one-way ANOVA with a Dunnett post-test, using WT hERG as control. *P* < 0.05 denoted by asterisks (*).

|  | WT | | Y652F | | Y652F1 | | Y652F2 | | F656F | | F656F1 | | F656F2 | |
| --- | --- | --- | --- | --- | --- | --- | --- | --- | --- | --- | --- | --- | --- | --- |
| V (mV) | τ (ms) | n | τ (ms) | n | τ (ms) | n | τ (ms) | n | τ (ms) | n | τ (ms) | n | τ (ms) | n |
| -150 | 14 ± 1 | 7 | 9 ± 1 * | 5 | 11 ± 1 | 7 | 13 ± 1 | 6 | 14 ± 0 | 7 | 17 ± 2 * | 4 | 14± 1 | 6 |
| -140 | 17 ± 1 | 7 | 11 ± 1 * | 5 | 13 ± 1 * | 7 | 17 ± 2 | 6 | 17 ± 0 | 7 | 21 ± 1 | 4 | 17 ± 1 | 6 |
| -130 | 23 ± 2 | 7 | 14 ± 1 * | 5 | 16 ± 1 * | 7 | 21 ± 2 | 6 | 22 ± 1 | 7 | 26 ± 1 | 4 | 22 ± 2 | 6 |
| -120 | 31 ± 2 | 7 | 18 ± 1 * | 5 | 21 ± 1 * | 7 | 29 ± 3 | 6 | 29 ± 1 | 7 | 35 ± 3 | 4 | 31 ± 2 | 6 |
| -110 | 47 ± 3 | 7 | 25 ± 2 * | 5 | 28 ± 2 * | 7 | 39 ± 4 | 6 | 40 ± 2 | 7 | 49 ± 4 | 4 | 47 ± 4 | 6 |
| -100 | 78 ± 8 | 7 | 36 ± 2 | 5 | 39 ± 3 | 7 | 59 ± 6 | 6 | 61 ± 3 | 7 | 72 ± 8 | 4 | 112 ± 26 | 6 |

**TABLE S3. Time constants of deactivation at potentials -150 to -100 mV of WT and all mutant constructs.** Values are mean ± standard error of the mean. Data are compared using a one-way ANOVA with a Dunnett post-test, using WT hERG as control. *P* < 0.05 denoted by asterisks (*).
